# Environmental-Nitrite-Enhanced Cyprinid Herpesvirus 2 Infection in Crucian Carp

**DOI:** 10.3390/vetsci13030244

**Published:** 2026-03-04

**Authors:** Qunlan Zhou, Qianhui Wang, Jun Qiang, Xiaodi Xu, Bo Liu, Shiqian Cao, Hualiang Liang

**Affiliations:** 1 Freshwater Fisheries Research Center, Chinese Academy of Fishery Sciences, No. 9 Shanshui East Road, Wuxi 214081, China; zhouql@ffrc.cn (Q.Z.); qiangjun@ffrc.cn (J.Q.);; 2 School of Fisheries and Life Science, Dalian Ocean University, Dalian 116023, China; 3 Wuxi Fisheries College, Nanjing Agricultural University, No. 69 Xuejiali, Wuxi 214128, China

**Keywords:** antioxidant enzyme, *Carassius auratus gibelio*, cytokine, NF-κB signal pathway, virus infection

## Abstract

The deterioration of water quality is associated with an increased disease risk, although the exact mechanism remains unclear. This study investigated the infection dynamics of cyprinid herpesvirus 2 (CyHV-2) in crucian carp (*Carassius auratus gibelio*) subjected to varying nitrite stress levels. A control group and three CyHV-2-infected groups exposed to nitrite concentrations of 0, 5, and 10 mg/L were set up. Results indicated that nitrite exposure caused a dose-dependent reduction in survival rates and decreased viral loads in the spleens of surviving fish. Nitrite stress elevated malondialdehyde (MDA) levels and glutathione peroxidase (GPx) activity, while reducing superoxide dismutase (SOD) and catalase (CAT) activities in the liver. Hepatic cytokine analysis revealed early peaks in tumor necrosis factor α (TNF-α) and interleukin 1β (IL-1β), alongside delayed response of interferon γ (IFN-γ) and interleukin 10 (IL-10), indicating impaired anti-inflammatory regulation. In the kidney, nitrite stress amplified immune gene expression, characterized by the upregulation of *tlr5* (*Toll-like receptor 5*) and *nf-κb* (*nuclear factor κB*) and the inhibition of *iκκβ* (*inhibitor of NF-κB kinase subunit β*), leading to prolonged NF-κB signaling. This was associated with a marked upregulation of *il-1β* and *il-8* (*interleukin 8*), alongside a delayed *ifn-γ* response. The combination of nitrite stress and CyHV-2 infection exacerbated oxidative damage and triggered a maladaptive immune response, thereby accelerating disease progression.

## Simple Summary

Intensified pond aquaculture leads to a deterioration in water quality, often leading to disease outbreaks. Although it is well established that poor water quality exacerbates disease risk, the mechanisms by which environmental stress contributes to disease outbreaks remain unclear. This study investigated viral infections in crucian carp (*Carassius auratus gibelio*) exposed to nitrite stress. The findings indicated that nitrite exposure caused a dose-dependent decrease in survival rates. This effect may be attributed to nitrite-stress-enhancing, virus-induced hepatic lipid peroxidation, caused premature hyperinflammatory responses, and disrupting anti-inflammatory regulation. In summary, environmental nitrite stress enhanced viral infections by enhancing oxidative damage, disrupting redox homeostasis, and triggering maladaptive immune responses, thereby accelerating disease progression.

## 1. Introduction

The intensification of aquaculture practices has led to a deterioration in water quality, especially in pond culture systems, resulting in the accumulation of harmful ammonia and nitrite levels. This environmental stress correlated with an increased incidence of diseases outbreaks among various aquatic species. Ammonia and nitrite are recognized to inhibit growth, to induce tissue damage, and to elevated mortality rates in fish. Exposure to these stressors during early life stages can reduce growth and survival rates in rare minnow (*Gobiocypris rarus*) [1] and cause tissue damage in various aquatic species, including false clownfish (*Amphiprion ocellaris*) [2] and *Macrobrachium amazonicum* [3]. Moreover, nitrite exposure caused oxidative stress and immune response in juvenile largemouth bass (*Micropterus salmoides*) [4] and silver carp (*Hypophthalmichthys molitrix*) [5]. Additionally, these stressors had been associated with inflammatory suppression and immune activation in the head kidney [6]. Poor water quality, like high ammonia and nitrite levels, increased viral infection sensitivity in largemouth bass [7], though the exact mechanism remains poorly understood.

Cyprinid herpesvirus 2 (CyHV-2), leading to herpes viral hematopoietic necrosis in crucian carp, causes high mortality and significant economic losses in intensive pond aquaculture. Initially identified in cultured *Carassius gibelio* Bloch in 2013 [8], CyHV-2 infection is characterized by symptoms such as body darkening, lethargy, hemorrhaging, and necrosis of the kidney and spleen. Subsequent studies have focused on developing rapid detection methods [9], exploring the infection mechanism [10,11], analyzing virus–host gene expression patterns during the infection [12,13,14], and evaluating potential vaccine candidates [15]. A latency mechanism for CyHV-2 has also been proposed, as viral DNA has been observed to persist in multiple tissues after acute infection, declining by 300 days post-infection, although the virus can be reactivated under temperature stress [16]. The potential for other environmental stressors, such as nitrite, to reactivate latent CyHV-2 remains uncertain.

The innate immune system functions as the primary defense against pathogens, with nuclear factor kappa B (NF-κB) crucial for antiviral responses by inducing the immune-related gene expression [17]. Activation of NF-κB signaling pathway reduced colonization of *Edwardsiella piscicida* and mortality in zebrafish (*Danio rerio*) [18], protected Nile tilapia (*Oreochromis niloticus*) from tilapia lake virus infection [19], and regulated inflammation in salmonid alphavirus infection in Atlantic salmon (*Salmo salar*) and rainbow trout (*Oncorhynchus mykiss*) [20]. Infectious hematopoietic necrosis virus infection in rainbow trout triggered oxidative stress and upregulated the immune-related genes like *nf-κb*, *tnf-α*, *il-1β*, *il-8*, etc. [21]. Meanwhile, CyHV-2 had been shown to activate the NF-κB signaling pathway, upregulating proinflammatory genes like *il-6*, *il-8*, *il-1β* and *tnf-α*, causing inflammation [22]. However, NF-κB activation could also promote viral gene transcription, potentially disrupting viral latency [17]. The role of NF-κB signaling pathway in the synergistic interaction between CyHV-2 infection and nitrite stress has not yet been elucidated.

The crucian carp (*Carassius auratus gibelio*) is widely cultured due to its robust immune system. In 2024, China’s national production surpassed 2.81 million tons, with Jiangsu Province contributing 0.56 million tons [23]. Since 2012, a novel viral disease, characterized by lethargy, anorexia, hemorrhage, pale gills, ascites, and splenorenal enlargement, has been identified to be caused by Cyprinid herpesvirus 2 (CyHV-2). This virus has become endemic in many aquaculture ponds, persisting in both water and sediment. Moreover, CyHV-2 can establish latency and reactivate under stress conditions [16]. This study examined CyHV-2 infection in crucian carp under varying nitrite stress levels. Hepatic antioxidant capacity and cytokine levels were detected to illustrate systemic inflammation. The kidney, as a major immune and hematopoietic organ in fish, was focused on profiling the transcriptional activation of immune-related genes to provide insights into immune signaling. It aimed to understand how environmental stress contributes to viral disease outbreaks and to support strategies, such as stringent nitrite control, to mitigate losses from viral disease in crucian carp aquaculture.

## 2. Materials and Methods

### 2.1. Ethics Statement

This research was conducted in accordance with the standards for scientific breeding and the utilization of fish established by the Animal Care and Use Committee of the Committee on the Ethics of Animal Experiments of the Freshwater Fisheries Research Center (LAECFFRC-2023-04-18).

### 2.2. Virus Preparation

**Figure 1:**
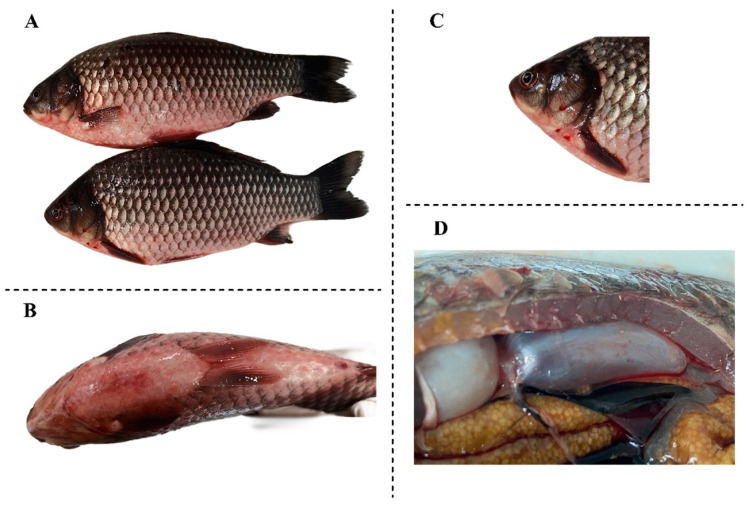
Diseased fish infected with Cyprinid herpesvirus 2. (**A**) The whole body of the diseased fish; (**B**) The head of the diseased fish; (**C**) The abdomen of the diseased fish; (**D**) The swim bladder of the diseased fish.

Diseased crucian carp with typical symptoms were collected from Sheyang, Yancheng city. The typical symptoms included varying degrees of hemorrhage (Figure 1A–C), hepatosplenomegaly (enlargement of liver and spleen), and petechial hemorrhages in the swim bladder (Figure 1D). Virus preparation followed the protocol described by Lu et al. [24]. In summary, infected spleen and kidney were aseptically collected from diseased fish, and then homogenized with sterile Dulbecco’s Modified Eagle Medium (DMEM, Gibco, Thermo Fisher Scientific, Waltham, MA, USA) with 1000 IU/mL penicillin and 1000 μg/mL streptomycin. The suspension was subjected to centrifugation at 3000 rpm for 15 min at 4 °C, and subsequently filtered through a 0.22 μm membrane filter. The suspension was inoculated onto a confluent monolayer culture of Koi fin (KF) cells (grown for approximately 24 h) in a 25 cm^2^ cell flask. The virus used in this study was a low-passage stock (passage 2 on KF cells). For virus propagation, cells were infected at a multiplicity of infection (MOI) of 0.1 and incubated at 25 °C. After adsorption for 1 h at 25 °C, fresh DMEM was added. The cells were harvested and frozen at −80 °C when more than 70% cytopathic effect was observed.

### 2.3. Absolute Viral Quantification

Inter-capsomeric triplex protein gene (GeneBank Accession No. EU349286) of CyHV-2 was chosen as target gene for absolute quantification. A plasmid containing the CyHV-2 inter-capsomeric triplex protein gene sequence was constructed using pUC57 as the vector. Primers were designed using online tool Primer-BLAST (Accessed on 20 May 2023. Available from: https://www.ncbi.nlm.nig.gov/tools/primer-blast) and synthesized by Shanghai Generay Biotech Co., Ltd. (Shanghai, China). The sequences of the primers were presented in Table 1: Forward: 5′-GCATGTGCGTCGACCTAGTA-3′ and Reverse: 5′-GTTCTTGACGCTCTGTCCGA-3′, and the amplicon was about 101 bp.

**Figure 2:**
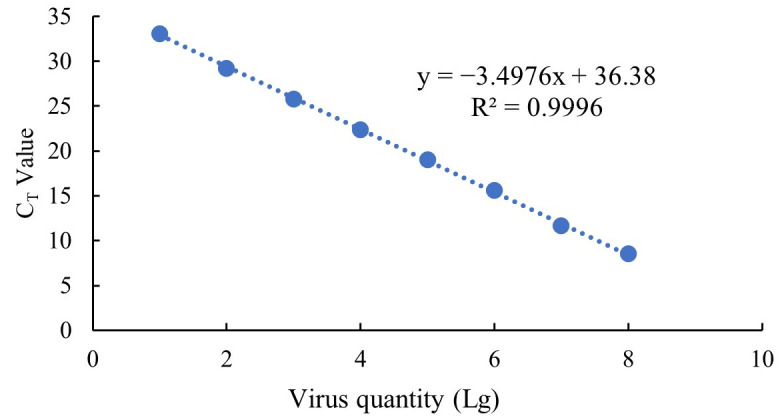
Absolute quantification standard curve.

The DNA plasmids concentration was detected at 260 nm using Nanodrop (Thermo Scientific, Waltham, MA, USA). After that, the plasmid concentration was adjusted to 1 × 10^8^ copies/μL, and then serially diluted 10-fold to 1 copy/μL. These diluted plasmids of varying concentrations were used as templates for Real-time Quantitative PCR (RT-qPCR) detection. RT-PCR was conducted using the TB Green Premix Ex Taq II kit (TaKaRa, Dalian, China) on a BioRad RT-PCR instrument (CFX96 Optics Module, Bio-Rad Laboratories, Inc., Singapore). Briefly, the 20 μL reaction mixture contained 2 μL DNA template, 0.8 μL of each primer (10 μmol/L), 10 μL of the premix and 6.4 μL ddH_2_O. The protocol was: 95 °C for 30 s; 39 cycles of 95 °C for 5 s and 60 °C for 30 s; 95 °C for 10 s; followed by a dissociation curve from 65 °C to 95 °C increasing by 0.5 °C every 5 s. A standard curve (Figure 2) was established based on the Ct value and 10-flod dilutions of plasmid containing inter-capsomeric triplex protein gene of CyHV-2.

### 2.4. Experimental Fish, Artificial Infection and Sample Collection

Healthy crucian carps were provided by the farm base of Freshwater Fisheries Research Center (Wuxi, China). After two weeks’ acclimation in the round polyvinyl chloride tanks (1000 height mm × 800 mm diameter, with a water volume about 350 L), a total of 256 crucian carps (body weight 300 ± 20 g) were randomly divided into four treatments with four replicates. Sixteen fish were cultured in one tank. Four different treatments were set. The first served as control group (Con) without CyHV-2 infection. The other three treatments were artificially infected with CyHV-2. Sodium nitrite was added into the tank to maintain the different nitrite stress. Then, the artificial infection treatments were as follows: artificial infected with CyHV-2 without nitrite stress (CyHV-2), artificial infected with CyHV-2 under 5 mg/L nitrite stress (5 mg/L +CyHV-2), and artificial infected with CyHV-2 under 10 mg/L nitrite stress (10 mg/L + CyHV-2). The whole experiment lasted one week. During the experimental period, fish were fed with commercial feed twice every day.

The detailed artificial viral infection process was as follows: Based on the results of preliminary experiments, the median lethal concentration (LC_50_) of CyHV-2 was determined to be 1 × 10^5^ copies/mL [10]. To investigate the impact of nitrite stress on CyHV-2 infection, a sublethal concentration of 1 × 10^4^ copies/mL was selected for injection challenge. Virus used for the injections were prepared according to previous descriptions: we detected the concentrations according to the absolute quantifications, and diluted it to 1 × 10^4^ copies/mL with DMEM. Fish were injected intraperitoneally with 1 mL per 100 g bodyweight, while control group received the same volume of blank KF cells with cell medium.

According to the previous study, 5 mg/L nitrite had no negative effects on juvenile crucian carp [27]. Based on our pre-experiment, nitrite stress level was set at 5 mg/L and 10 mg/L, respectively. Nitrite solution was prepared by dissolving NaNO_2_ in 5 L distilled water to make a stock solution and then diluted step by step. Nitrite concentration was measured by the N-1naphthylethylenediamine photometric method [28], and a quarter of the water was regularly changed every 24 h to adjust the nitrite concentration to the initial. During the experiment, water was aerated. Water quality was detected every morning. Water temperature was measured using a mercury thermometer, pH was determined with a pH meter, and dissolved oxygen and ammonia nitrogen levels were assessed with YSI Multi-parameter Water Quality Monitor (Xylem Inc., Yellow Springs, OH, USA). Throughout the whole experimental period, water temperature was maintained at 24.5 ± 1.0 °C, pH between 7.5 and 7.8, dissolved oxygen ≥ 8 mg/L, and ammonia ≤ 0.2 mg/L.

All infected fish were continuously observed for 6 days post-infection, with mortality record daily. Concurrently, liver, spleen and kidney tissues were aseptically collected at 0 h post-infection (pre-infection baseline) and at 1-, 2-, 3-, 4-, 5-, and 6-day post-infection (dpi). Four biological replicates (individual fish) per treatment were sampled at each time point. Samples were stored at −80 °C for later analysis.

### 2.5. Virus Load Detection in the Infected Fish

Approximately 0.1 g of spleen from infected crucian carps was subjected to DNA extraction using Ezup Column Viral DNA Extraction Kit (Sangon Biotech, Shanghai, China). The concentration was measured with a Nanodrop (Thermo Scientific, Waltham, MA, USA) and adjusted to 10 ng/μL. Absolute quantification of viral copy number in the spleen was performed based on the previous absolute viral detection according to the standard curve (Figure 2) based RT-qPCR assay.

### 2.6. Antioxidant Enzyme Activities and Cytokines

The liver was chosen to detect antioxidant enzyme activities and cytokines following viral and nitrite stress. About 0.1 g liver sample was homogenized with 0.9 mL of 0.85% NaCl saline. After centrifuging at 4000 rpm for 10 min at 4 °C, the supernatant was gotten. Protein concentration was measured with a Bradford assay, and the activities of superoxide dismutase (SOD), catalase (CAT), glutathione peroxidase (GPx), and the malondialdehyde (MDA) content were assessed using commercial kits from Nanjing Jiancheng Bioengineering Institute (Nanjing, China).

Cytokines, including tumor necrosis factor α (TNF-α), interleukin 1β (IL-1β), interleukin 8 (IL-8), interferon γ (IFN-γ), transforming growth factor β (TGF-β), and interleukin 10 (IL-10), were measured using the method by Muhammad [29] with commercial kits from Shanghai Enzyme-linked Biotechnology Co., Ltd. (Shanghai, China). The process involved forming an antibody-antigen-enzyme-antibody complex with cytokines and detecting it spectrophotometrically at 450 nm.

### 2.7. Relative Expression of Genes Related with Immunity

The kidney, as a major immune organ in fish, was analyzed for immune-related gene expression. RNA was extracted with RNAiso (Takara, Dalian, China) and assessed for quality and quantity using a NanoDrop spectrophotometer (Thermo Scientific, Waltham, MA, USA). cDNA synthesis was performed with the ExScript^TM^ RT-PCR kit (Takara, Dalian, China) and amplified using the TB Green^®^ Premix Ex Taq^TM^ II Kit (Takara, Dalian, China) on a CFX96 RT-PCR system (Bio-Rad, Hercules, CA, USA). Primers were provided by Shanghai Gene-ray Biotech Co. Ltd. (Shanghai, China) (Table 1). Reaction mixture and reaction conditions were as described by the kit’s manufacture. *β-actin* was chosen to be housekeep gene. Gene expression data were calculated with the 2^−∆∆Ct^ method [30].

### 2.8. Statistical Analysis

Data were analyzed using SPSS v19 (Chicago, IL, USA) and GraphPad Prism 8. Two-way analysis of variance (ANOVA) assessed treatment and infection time effects, while one-way ANOVA evaluated differences among treatments or times. Turkey’s tests determined significance at *p* < 0.05. Figures, including the survival curves and histograms, were created with GraphPad Prism 8.

## 3. Results

### 3.1. Survival and Viral Load in Survivor Post-Challenge

**Figure 3:**
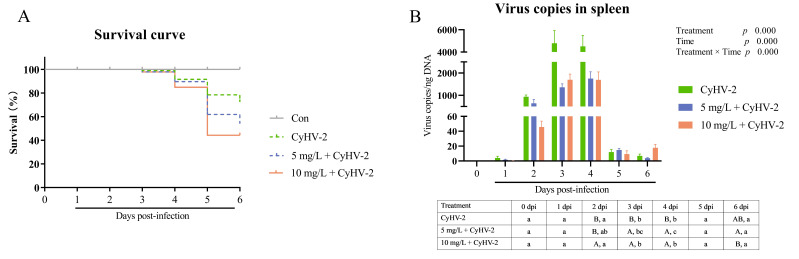
Survival rate and splenic viral load in crucian carp after CyHV-2 challenge. (**A**) survival rate after artificial CyHV-2 infection; (**B**) virus copies in spleen of survivor fish. Lowercase letters denoted significant differences over time within the same treatment after Turkey’s test (*p* < 0.05); while uppercase letters indicated significant differences between treatments at the same time (*p* < 0.05) (*n* = 4).

The survival of crucian carp following CyHV-2 challenge was shown in Figure 3A. The control group exhibited no mortality, maintaining a 100% survival rate. In contrast, artificial challenge with CyHV-2 resulted in sporadic mortality commencing at 3 dpi, with a peak mortality period during 4–5 dpi, after which survival rates stabilized by 6 dpi. The final survival rate in the CyHV-2 only group (positive control) was 72.41%. Notably, co-exposure to nitrite stress significantly exacerbated mortality in a dose-dependent manner, reducing survival rates to 54.07% and 44.27% at nitrite concentrations of 5 mg/L and 10 mg/L, respectively (Figure 3A).

The dynamics of splenic viral load in survivors were presented in Figure 3B, revealing a consistent pattern across infected groups: viral loads began to rise significantly at 2 dpi, peaked at 3–4 dpi, coinciding with peak mortality, and then markedly declined by 5–6 dpi. Two-way ANOVA revealed that both environmental stress and infection duration had extremely significant effects on viral loads (*p* < 0.001), with a significant interaction between environmental treatment and infection duration (*p* < 0.001). Despite the increased mortality observed, the viral loads in fish that survived under nitrite stress were significantly lower than those in the CyHV-2 group during the peak replication phase (3–4 dpi) (*p* < 0.05).

### 3.2. Hepatic Antioxidant Response Post-Challenge

**Figure 4:**
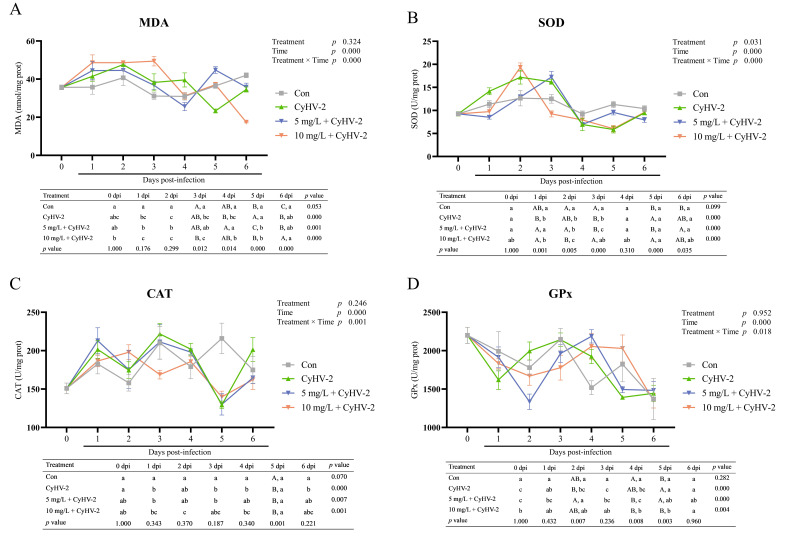
Antioxidant ability of surviving crucian carp after CyHV-2 challenge. (**A**) MDA, malondialdehyde; (**B**) SOD, superoxide dismutase; (**C**) CAT, catalase; (**D**) GPx, glutathione peroxidase. Lowercase letters denoted significant differences over time within the same treatment after Turkey’s test (*p* < 0.05); while uppercase letters indicated significant differences between treatments at the same time (*p* < 0.05) (*n* = 4).

The hepatic antioxidant response was assessed by measuring MDA content and the activities of key antioxidant enzymes, including SOD, CAT and GPx (Figure 4). A two-way ANOVA revealed that the duration of infection had an extremely significant effect on all measured parameters (*p* < 0.001), while nitrite co-exposure significantly influenced only SOD activity (*p* < 0.05). Significant interactive effects between treatment and infection duration were observed for all parameters (*p* < 0.05). In the control group, all metrics maintained stable throughout the experiment (*p* > 0.05).

CyHV-2 infection alone induced significant time-dependent oxidative stress. Hepatic MDA content, serving as an indicator of lipid peroxidation, increased rapidly shortly after infection, peaking at 2 dpi, followed by a progressively decline from 3 dpi onward (*p* < 0.001) (Figure 4A). SOD activity increased slowly, peaking at 2 dpi, and then decreased sharply, reaching its lowest at 5 dpi (*p* < 0.001) (Figure 4B). CAT activity fluctuated significantly, peaking at 1, 3, and 6 dpi (*p* < 0.001) (Figure 4C). GPx activity initially decreased, subsequently increased, and then significantly declined once more (*p* < 0.001) (Figure 4D).

With co-exposure to nitrite, CyHV-2 infection intensified and modified the oxidative stress profile. Although the overall kinetic pattern of MDA was similar, the group exposed to 10 mg/L nitrite exhibited higher and more sustained MDA peaks during 1–3 dpi, with the lowest MDA level occurring later than in the group exposed to CyHV-2 alone (Figure 4A). Nitrite stress also disrupted the temporal coordination of the antioxidant system. The SOD activity peak occurred earlier under 10 mg/L nitrite stress, followed by a deeper suppression (Figure 4B). A sharp decline in CAT activity was specifically observed at 3 dpi in the 10 mg/L nitrite group, coinciding with persistently high MDA levels (Figure 4C). In contrast, GPx activity was significantly upregulated in the high nitrite group, suggesting a compensatory response specifically targeting peroxide accumulation induced by nitrite exposure (Figure 4D).

### 3.3. Hepatic Cytokine Profiles Post-Challenge

**Figure 5:**
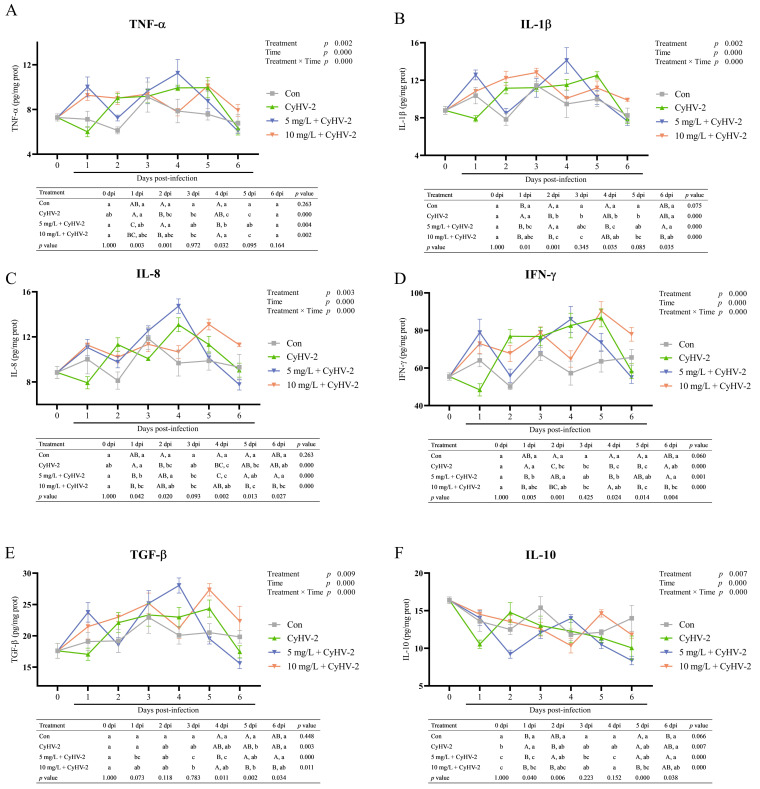
Hepatic cytokines levels of surviving crucian carp after CyHV-2 challenge. (**A**) TNF-α, tumor necrosis factor α; (**B**) IL-1β, interleukin 1β; (**C**) IL-8, interleukin 8; (**D**) IFN-γ, interferon γ; (**E**) TGF-β, transforming growth factor β; (**F**) IL-10, interleukin 10. Lowercase letters denoted significant differences over time within the same treatment after Turkey’s test (*p* < 0.05); while uppercase letters indicated significant differences between treatments at the same time (*p* < 0.05) (*n* = 4).

The hepatic cytokine response was profoundly influenced by CyHV-2 infection and nitrite exposure (Figure 5). Two-way ANOVA analysis revealed that both the treatment and the duration of infection, as well as their interaction, exerted highly significant effects (*p* < 0.01) on all measured cytokines, including TNF-α, IL-1β, IL-8, IFN-γ, TGF-β, and IL-10. In the control group, the hepatic cytokine levels remained stable during the experimental period (*p* > 0.05).

In fish challenged solely with CyHV-2, most proinflammatory cytokines (TNF-α, IL-1β, IL-8) (Figure 5A–C) and IFN-γ (Figure 5D) exhibited a moderate decrease at 1 dpi, but subsequently maintained elevated levels from 2 to 5 dpi, reaching their peak at 4 or 5 dpi. In contrast, the anti-inflammatory cytokine IL-10 showed a declining trend beginning at 2 dpi (Figure 5F).

In the group subjected to CyHV-2 infection under 5 mg/L nitrite stress, hepatic cytokines (Figure 5A–E), except for IL-10, exhibited an initial increase at 1 dpi, followed by a decline at 2 dpi, and subsequently rose to reach their peak levels at 4 dpi before decreasing (*p* < 0.001). In contrast, during CyHV-2 infection under 10 mg/L nitrite stress, there was a consistent elevation in the cytokine levels of TNF-α, IL-1β and TGF-β from 1 to 3 dpi, followed by fluctuations over the subsequent three days (*p* < 0.001) (Figure 5A,B,E). Hepatic IL-8 and IFN-γ levels varied post-CyHV-2 infection under 10 mg/L nitrite stress, peaking on day 5 dpi (*p* < 0.001) (Figure 5C,D). Regarding IL-10, a noticeable declining trend was observed following CyHV-2 challenge under both 5 mg/L and 10 mg/L nitrite conditions until 4 dpi, after which levels increased to peak on 5 dpi (*p* < 0.001) (Figure 5F).

Cross-group comparisons further underscored the immunomodulatory effects of nitrite. At 1 dpi, both nitrite-exposed groups displayed significantly higher cytokine levels than the CyHV-2 only group, indicating premature immune activation. The 5 mg/L + CyHV-2 group displayed significantly elevated cytokine levels at 4 dpi, whereas the 10 mg/L + CyHV-2 group maintained high levels of IL-8, IFN-γ, and TGF-β at the later stages (5–6 dpi), suggesting a dysregulated and unresolved inflammatory response at the higher nitrite concentration.

### 3.4. Immune-Related Gene Expression in Kidney Post-Challenge

**Figure 6:**
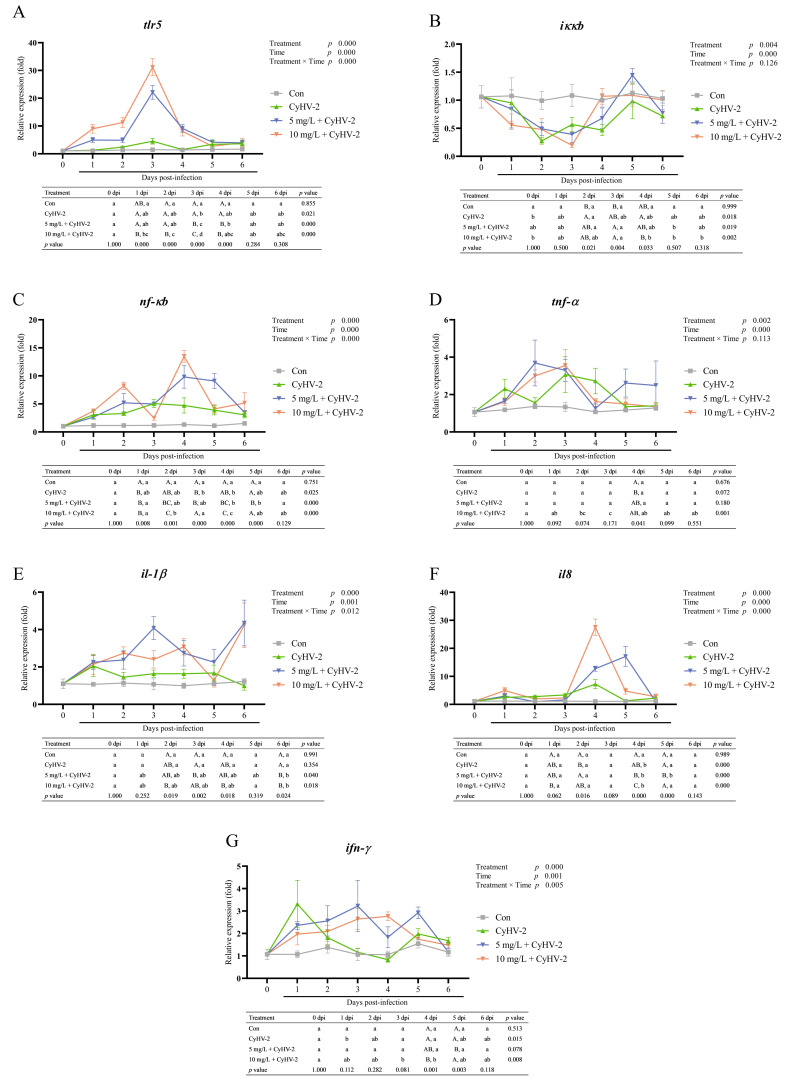
Relative expression of genes related to immunity in kidney of surviving crucian carp after CyHV-2 challenge. (**A**) *tlr5*, Toll-like receptor 5; (**B**) *iκκβ*, inhibitor of nuclear factor Kappa-B kinase subunit beta; (**C**) *nf-κb*, nuclear factor Kappa-light-chain-enhancer of activated B cells; (**D**) *tnf-α*, tumor necrosis factor α; (**E**) *il-1β*, interleukin 1β; (**F**) *il-8*, interleukin 8; (**G**) *ifn-γ*, interferon γ. Lowercase letters denoted significant differences over time within the same treatment after Turkey’s test (*p* < 0.05); while uppercase letters indicated significant differences between treatments at the same time (*p* < 0.05) (*n* = 4).

The immune-related gene expression in the kidney was highly modulated by CyHV-2 infection and nitrite stress (Figure 6). Two-way ANOVA results revealed significant effects of both treatment and infection duration (*p* < 0.01), as well as significant interactive effects for all genes except *iκκb* and *tnf-α* (*p* < 0.01). In contrast, gene expression in the control group remained stable across all time points (*p* > 0.05).

CyHV-2 infection alone markedly upregulated the transcription of key immune genes, including *tlr5*, *nf-κb*, *il8* and *ifn-γ*, while suppressing the negative regulator *iκκb* (Figure 6A–C,F,G). The expression dynamics were time-dependent: *ifn-γ* was induced early at 1 dpi, *iκκb* was inhibited at 2 dpi, *tlr5* and *nf-κb* were activated at 3 dpi, and *il-8* peaked at 4 dpi.

Concurrent exposure to nitrite amplified and altered these transcriptional responses. Specifically, nitrite stress enhanced the activation of proinflammatory genes and those in the NF-κB pathway (Figure 6A–C). The expression levels of *tlr5* (at 3 dpi) and *nf-κb* (at 2 and 4 dpi) were elevated higher in nitrite-exposed groups than to those infected with CyHV-2 alone. Concurrently, the suppression of *iκκb* was significantly intensified, particularly at 3 dpi in the 10 mg/L group, suggesting impaired negative feedback on NF-κB signaling pathway.

The expression of cytokine genes exhibited differential regulation (Figure 6E–G). Specifically, the relative expression of *il-1β* was remarkably upregulated under nitrite stress at 2–4 dpi. In contrast, *il-8* expression was further amplified by nitrite exposure at 4 dpi. Meanwhile, *ifn-γ* expression was initially suppressed during the mid-phase of infection, followed by a compensatory increase in the 10 mg/L group at later stage (4–5 dpi).

## 4. Discussion

In this study, a CyHV-2 infection model was successfully established, which was evidenced by the significant decreased survival rate of 72.41% in the challenged group, compared to the 100% survival rate observed in the negative controls. Notably, survival rates were further reduced to 54.07% and 44.27% at 5 and 10 mg/L nitrite levels, respectively. These survival rates were significantly lower than those in the virus-only group, indicating a dose-dependent relationship between increased nitrite concentration and accelerated disease outbreak. This finding underscores that environmental stress is a critical modulator of disease outcomes, aligning with previous research on juvenile largemouth bass, which found nitrate concentrations related with mortality of virus and the viral load [7]. Additionally, ammonia nitrogen accumulation increased susceptibility of *Litopenaeus vannamei* to *Vibrio parahaemolyticus* [31]. As ectothermic vertebrates, fish are very sensitive to environmental changes, particularly during pathogen infections [32]. Similar observations reported in sockeye salmon (*Oncorhynchus nerka*) [33]. Acute nitrite exposure would proliferate pathogenic *Photobacterium* and drive mortality in Pacific white shrimp (*Penaeus vannamei*) [34].

We recognize several methodological considerations that may influence the interpretation of our findings. The primary aim of this study was to explore the mechanism of CyHV-2 infection in crucian carp under environmental nitrite stress. Consequently, our experimental design prioritized viral challenge experiments across varying nitrite concentrations, omitting a nitrite-only control group. While this approach is consistent with our research objectives, it complicates the disentanglement of nitrite’s direct effects from its interactions with the virus. Nonetheless, prior studies have shown that exposure to 5 mg/L nitrite in crucian carp for 30 days [27] or acute exposure to 10 mg/L nitrite in grass carp (*Ctenopharyngodon idella*) [35] does not cause mortality. This suggests that the decreased survival observed in our co-exposure groups is likely attributable to synergistic mechanisms, rather than nitrite toxicity alone, although an additive effect cannot be entirely ruled out. Furthermore, without histopathological examination, the structural correlates of the observed organ dysfunction remain uncharacterized. Future studies should incorporate detailed histopathological assessments to further elucidate these findings.

The temporal dynamics of viral load have provided critical insights into disease progression. The observation that the viral load speak (3–4 dpi) preceded the peak mortality (4–5 dpi), suggested that host mortality is more likely a consequence of immunopathological damage and tissue injury following peak viremia, rather than solely due to the initial viral replication. This pattern is consistent with infections caused by other piscine viruses such as koi herpesvirus (CyHV-3) [36] and infectious salmon anemia virus (ISAV) [37]. In CyHV-3 infected carp at 22 °C, virus expression was abundant and correlated with tissue damage, clinical disease, and mortality [36]. Similarly, in Atlantic salmon (*Salmo salar*) infected with ISAV, viral loads dropped markedly during the late phase of infection [37]. In farmed Atlantic salmon, rapid viral replication led to extensive cellular damage before the host’s immune system could develop an effective response [38]. The substantial decline in viral load among survivors by 5–6 dpi suggested the onset of effective immune clearance.

An interesting finding in our study was the significant reduction in viral load under nitrite stress, despite a concurrent increase in mortality. It meant that a low viral load did not necessarily correlate with a mild disease outcome. We hypothesize that under the combined stressors of nitrite exposure and virus infection, the fish immune system became overloaded, resulting in a lower threshold for viral impact compared to normal status. That was to say, under environmental stress, a viral load that would typically cause morbidity may instead lead to mortality in fish. Nitrite stress has been shown to cause gill damage and impair osmoregulatory and respiratory functions, as documented in grass carp [35], false clownfish (*Amphiprion ocellaris*) [2], and yellow catfish (*Pelteobagrus fulvidraco*) [39]. In summary, nitrite stress might cause oxidative stress and immunosuppression, potentially facilitating the viral invasion or overwhelm of the host’s immune defense, thereby triggering more severe disease outbreaks. Further research is needed to uncover the detailed mechanisms underlying these observations.

This study demonstrated that CyHV-2 infection elicited significant time-dependent oxidative stress in the liver, characterized by an early peak in lipid peroxidation, as indicated by MDA levels, followed by a dysregulated antioxidant enzyme response. The subsequent decline in MDA, particularly in the group infected solely with CyHV-2, may have resulted from the activation of antioxidant defenses. This pattern is similar with that observed in grass carp reovirus infection in grass carp, where oxidative damage led to cell autophagy [40].

Concurrent exposure to nitrite during CyHV-2 infection, particularly at a concentration of 10 mg/L, profoundly exacerbated oxidative injury. Nitrite stress intensified the oxidative damage, leading to elevated and prolonged levels of MDA and a delayed return to baseline conditions. The suppression of SOD and CAT activities during the late phase of infection phase (in 4 dpi and 5 dpi) indicated a failure of the hepatic antioxidant system, leading to persistent oxidative injury. The data suggested that nitrite stress not only amplified the severity of lipid peroxidation but also altered its temporal progression and disrupted the kinetics of key antioxidant enzymes. This prolonged oxidative damage aligned with the notably reduced survival rate in this group. Previous studies have demonstrated that nitrite exposure caused oxidative stress in various fish species. In yellow catfish, it reduced SOD, CAT and GPx activities while increasing MDA levels [39]. In zebrafish, nitrite exposure reduced antioxidant capacity and glutathione, resulting in mitochondrial damage [41,42]. In spotted seabass (*Lateolabrax maculatus*), nitrite decreased CAT and SOD activities in the gills and raised MDA content [43]. Similar were observed in grass carp [35], bighead carp (*Aristichthys nobilis*) [44], and tilapia (*Oreochromis niloticus*) [45]. Overall, nitrite exposure exacerbated and altered the timing and extent of lipid peroxidation triggered by CyHV-2. Additionally, as nitrite concentration increased, there was a more pronounced peak and a more prolonged elevation in hepatic MDA levels, alongside a significant suppression of SOD and CAT activities, demonstrating a dose-dependent effect. High nitrite levels caused both an earlier onset of severe damage and a more substantial later suppression of peroxidation.

Beyond merely intensifying oxidative damage of CyHV-2 infected fish, nitrite stress critically disrupted the coordination and timing of the hepatic antioxidant defense system. Despite a peak in SOD activity at 2 dpi, MDA levels continued to rise, indicating an inadequate antioxidant response. Similar results reported in common carp infected with *Aeromonas hydrophila* [46]. CAT activity was sharply reduced at 3 dpi in the 10 mg/L nitrite group, allowing hydrogen peroxide to accumulate and cause ongoing lipid peroxidation. Moreover, nitrite altered the temporal dynamics of enzyme, with an earlier SOD peak indicating a brief compensatory response and sustained GPx upregulation possibly adapting to nitrite-derived peroxides. The lack of a clear inverse relationship between antioxidant enzyme activities and MDA levels might mean a significant disruption in redox balance. This imbalance, characterized by an overwhelmed and desynchronized antioxidant system, suggested severe combined stress. Thus, the combined hepatotoxicity of CyHV-2 and nitrite stress resulted not only from increased oxidative damage but also from the host’s antioxidant system failing to respond effectively and promptly.

It should be noted that the absence of a control group exposed solely to nitrite, which limited the ability to distinguish between the effects of nitrite and its synergistic interaction with viral infection. Nonetheless, existing research had shown that chronic exposed to 5 mg/L nitrite for 30 days altered the hepatic SOD and CAT activities in crucian carp with fluctuations in glutathione levels, which was similar without obvious difference with those observed at 0 and 2.5 mg/L nitrite concentrations [27]. It indicated that nitrite alone really induced oxidative stress, albeit not prominently. Additionally, acute exposure to 10 mg/L nitrite in grass carp has been shown to induce oxidative damage [38]. However, it is noteworthy that neither the long-term exposure of crucian carp to 5 mg/L nitrite nor the acute exposure of grass carp to 10 mg/L nitrite resulted in mortality. In contrast, a significantly reduced survival rate was observed in crucian carp infected with a virus and exposed to nitrite stress in this study. This finding further supports the observation that oxidative stress induced by environmental factors exacerbates mortality during viral infection, indirectly suggesting that environmental oxidative stress contributed to the impairment of the fish’s antioxidant system.

It was found that nitrite stress significantly altered the immune response to CyHV-2 infection, affecting cytokine profiles in a dose-dependent manner in this study. Notably, nitrite exposure did not merely enhance inflammation but also modified the timing of the immune response. Similar results observed in silver carp (*Hypophthalmichthys molitrix*), where nitrite exposure significantly altered cytokines TNF-α and IL-1β levels [6]. A key finding in this study was that nitrite stress prematurely activated the innate immune system, with proinflammatory cytokines like TNF-α and IL-1β rising by 1 dpi, prior to the peak of viral replication. This suggests activation of the NF-κB pathway. The early cytokine surge, like findings in yellow catfish (*Pelteobagrus fulvidraco*), where nitrite exposure caused the cytokines release including TNF-α and IL-8 [39], highlighting a shift in the timing of the immune response. The observed reduction in cytokine levels suggested a weakened cellular immunity response, thereby increasing the risk of infection under stress conditions [47].

Furthermore, our data showed a disruption in anti-inflammatory regulation, characterized by an early and sustained cytokine IL-10 decline, especially in the 10 mg/L group, indicating a reduction in inflammation. High TNF-α and IL-1β levels, along with a low IL-10 in the high nitrite group, inevitably created an environment of uncontrolled inflammation. It led to more severe tissue damage, particularly when combined with concurrent oxidative stress, evidenced by elevated MDA contents and suppressed SOD and CAT activity.

The immunomodulatory effects of nitrite over CyHV-2 infected fish were further elucidated by its dose-dependent impact, which could be proved by an earlier and more exaggerated proinflammatory cytokine response (TNF-α, IL-1β) and a delayed anti-inflammatory response (IL-10) with increasing nitrite concentrations. In the 5 mg/L group, immunosuppression was followed by a pronounced cytokine peak at 4 dpi, suggesting a delayed yet potentially adaptive inflammatory response aligned with efforts to clear the virus. Conversely, exposure to 10 mg/L nitrite induced a maladaptive and dysregulated immune response, such as an early and sustained elevation of proinflammatory cytokines, along with delayed peaks of IFN-γ and IL-8. This temporal disjunction in the immune response, specifically the delayed surge in IFN-γ, likely reflected a failed attempt at viral clearance, thereby contributing to chronic inflammation and decreased survival rates under high nitrite stress. The premature and prolonged inflammatory response was not only dysregulated but also served as a driver of immunopathological damage. Such a cytokine storm is known to cause tissue injury and organ dysfunction.

The NF-κB pathway plays a pivotal role in the expression of antiviral genes in eukaryotic cells [48]. Our study showed that CyHV-2 infection activated the NF-κB pathway, as evidenced by upregulation of *tlr5*, *nf-κb*, and proinflammatory genes like *il-8*, consistent with previous findings [22]. Additionally, *ifn-γ*, *il-10*, *il-12* expression was found to be upregulated in goldfish fin cells co-culture with CyHV-2 [49]. A novel and significant finding in our study was that nitrite stress significantly amplified activation and disrupted regulatory feedback mechanisms in a dose-dependent manner. This was proved by the enhanced upregulation of immune-related genes (e.g., *tlr5*, *nf-κb*, and *il8*) with increasing nitrite concentrations. The enhanced activation of *tlr5* and *nf-κb*, along with significant suppression of *iκκβ*, indicated sustained dysregulation of NF-κB signaling. The deepest suppression of *iκκβ* at 3 dpi in the 10 mg/L group suggested a failure in the feedback loop typically responsible for resolving NF-κB activation. Concurrently, elevated proinflammatory protein levels in the liver and robust transcriptional upregulation of genes within the NF-κB pathway in the kidney indicated a coordinated yet organ-specific immune activation. This dysregulated NF-κB activation may be the main cause of reduced survival in fish infected with CyHV-2 under nitrite stress.

The interaction between nitrite exposure and the duration of infection significantly disrupted the timing of the immune response. Initially, nitrite exposure suppressed *iκκβ* and increased *nf-κb*, setting the system to hyperinflammatory condition. In the mid-phase, nitrite further amplified the virus-induced *il-8* response, potentially exacerbating tissue damage. Meanwhile, nitrite delayed *ifn-γ* upregulation, thereby impairing timely antiviral defenses, which was crucial for clearing the virus. A late increase in *ifn-γ* in the 10 mg/L group was likely ineffective, as evidenced by high mortality rates. This dysregulation of inflammation response and impaired antiviral immunity was aligned with findings from zebrafish studies, in which nitrite exposure was observed to suppress the splenic expression of *il-1β* and *ifn-γ* [42]. During the initial week of exposure to *Aeromonas hydrophila*, tilapia (*Oreochromis niloticus*) showed reduced levels of the proinflammatory cytokines *tnf-α* and *il-1β*, whereas the anti-inflammatory cytokine *il-10* was highly upregulated before subsequently declining [50]. The deleterious effects of nitrite and CyHV-2 stemmed from NF-κB pathway disruption. Nitrite stress disrupted immune homeostasis, causing excessive inflammation and weak antiviral responses, thereby accelerating disease progression and increasing mortality.

It was demonstrated that nitrite stress exacerbated CyHV-2 induced mortality and led to dysregulated immune responses, particularly through sustained NF-κB activation in the present study. Notably, CyHV-2, like many herpesviruses, can enter a latent state and reactivated under stress conditions [16]. In the context of our findings, nitrite-induced oxidative stress and NF-κB hyperactivation may create a permissive environment for viral reactivation. NF-κB is a key regulator of not only inflammatory responses but also viral gene transcription, and its prolonged activation could potentially disrupt viral latency [17]. For example, the activation of NF-κB has been implicated in the reactivation of latent human immunodeficiency virus 1 (HIV-1). The marked suppression of iκκβ, a negative regulator of NF-κB, under conditions of high nitrite exposure, suggested impaired feedback control, potentially facilitating viral reactivation. Although the present study did not directly assess latent virus reactivation, the immunological and oxidative disturbances observed are aligned with mechanisms known to trigger herpesvirus reactivation [17]. Future studies should investigate how nitrite stress may reactivate latent CyHV-2 in recovered or subclinical infected fish, which would further elucidate the environmental drivers of CyHV-2 outbreaks in aquaculture settings.

Besides its immunomodulatory role, the kidney in freshwater teleost is crucial for osmoregulation [51,52], continuously producing hypotonic urine to counteract passive water influx. The renal transcriptional activation of inflammatory genes observed in this study suggested possible structural or functional impairments induced by CyHV-2 infection. Nitrite, as an oxidative stressor, may further aggravate osmoregulatory dysfunction. Although the present study did not assess renal excretory performance or histopathology, previous studies have shown that exposure to varying salinity or alkalinity can disrupt immune and oxidative homeostasis and may alter kidney structure [53,54]. Such disruption in homeostasis likely exacerbates the physiological decline in virus-infected fish under nitrite stress. Future research incorporating renal histopathology and plasma osmolality measurements would be conducted in determining whether nitrite-mediated dysregulation of osmoregulation contributes to mortality during CyHV-2 outbreaks.

## 5. Conclusions

Nitrite stress enhanced CyHV-2 infection in crucian carp by reducing survival rates and increasing oxidative stress within the liver. This stressor triggered premature hyperinflammatory response while simultaneously delaying adaptive immune responses, leading to uncontrolled inflammation and accelerated disease progression. These findings underscore the critical importance of controlling nitrite concentrations in aquaculture environments to mitigate the risk of disease outbreaks.

## Figures and Tables

**Table 1:** Real-time PCR primers’ sequences.

Gene	Primer Sequence (5′-3′)	Amplification Efficiency (%)	Accession No./Reference
*CyHV-2*	F: GCATGTGCGTCGACCTAGTA R: GTTCTTGACGCTCTGTCCGA	-	EU349286
*nf-κb*	F: GCCACTAAATCCACCACATC R: AACCCAAGCAGTTCACATACA	109.27	KM393205.1 [22]
*iκκβ*	F: GCCAGCAATGGAAGGTCAT R: GCTCAGCGACAAGAATAAAGG	97.59	NM001123265.1 [22]
*tlr5*	F: AATCCAGCATACAATGTGAGG R: TCCATCCACAAGTTTAGCAAT	106.25	KX759644 [25]
*tnf-α*	F: CATTCCTACGGATGGCATTTACT R: CCTCAGGAATGTCAGTCTTGCAT	108.23	EU069818.1 [22]
*il-1β*	F: GGGAGCGGGACTATATGCTG R: TGCGTAACGACACAGGTGAA	100.05	NM212844.2 [22]
*il-8*	F: CTGAGAGTCGACGCATTGGA R: GTGCAGTAGGGTCCAGACAG	108.42	KC184490.1 [22]
*ifn-γ*	F: CTACGGGTCCTGAAAGACTT R: GCCTGGGAAGTAGTTTTCTC	114.32	[26]
*β-actin*	F: CACTGTGCCCATCTACGAG R: CCATCTCCTGCTCGAAGTC	105.15	AB039726.2 [22]

Note: *CyHV-2*, inter-capsomeric triplex protein gene of cyprinid herpesvirus 2; *nf-κb*, nuclear factor Kappa-light-chain-enhancer of activated B cells; *iκκβ*, inhibitor of nuclear factor Kappa-B kinase subunit beta; *tlr5*, Toll-like receptor 5; *tnf-α*, tumor necrosis factor α; *il-1β*, interleukin 1β; *il-8*, interleukin 8; *ifn-γ*, interferon γ; *β-actin*, Beta-actin.

## Data Availability

The original contributions presented in this study are included in the article. Further inquiries can be directed to the corresponding author.

## Abbreviations

The following abbreviations are used in this manuscript:

ANOVA	Analysis of Variance
CAT	Catalase
CyHV-2	Cyprinid Herpesvirus 2
CyHV-3	Koi Herpesvirus
dpi	Day post-infection
GPx	Glutathione Peroxidase
HIV-1	Human immunodeficiency virus 1
IFN-γ	Interferon γ
Iκκβ	Inhibitor of Nuclear Factor Kappa B Kinase Subunit Beta
IL-1β	Interleukin 1β
IL-8	Interleukin 8
IL-10	Interleukin 10
ISAV	Infectious Salmon Anemia Virus
MDA	Malondialdehyde
NF-κB	Nuclear Factor Kappa B
RT-qPCR	Real-time Quantitative PCR
SOD	Superoxide Dismutase
TGF-β	Transforming Growth Factor β
TLR5	Toll-like Receptor 5
TNF-α	Tumor Necrosis Factor α
